# Advertising Payments to News Websites That Publish Health Misinformation

**DOI:** 10.1001/jamanetworkopen.2026.5068

**Published:** 2026-04-01

**Authors:** Neeraj G. Patel, Reshma Ramachandran, Joseph S. Ross

**Affiliations:** 1 Yale School of Medicine, New Haven, Connecticut; 2 Section of General Medicine, Department of Internal Medicine, Yale School of Medicine, New Haven, Connecticut; 3 Yale Collaboration for Regulatory Rigor, Integrity, and Transparency, Yale School of Medicine, New Haven, Connecticut; 4 Department of Health Policy and Management, Yale School of Public Health, New Haven, Connecticut

## Abstract

This cross-sectional study estimates advertising spending on the part of government and health organizations to news websites that publish health misinformation.

## Introduction

Exposure to health misinformation, defined in a US Surgeon General Advisory as incorrect or misleading health information according to the best available evidence at the time, has increasingly spread through social media platforms, online forums, and smaller media outlets.^1^ Although several strategies focus on mitigating its reach, there has been recent interest in curbing misinformation at its source. Prior reports have suggested that misinformation websites are motivated by advertising revenue^2^ and that well-known brands frequently place advertisements on misinformation-spreading websites.^3,4^ Research has not focused on advertising by governments or health organizations, whose missions may be inconsistent with supporting misinformation-spreading websites. We characterized estimated payments made by government and health organizations to place advertisements on news websites identified for publishing health misinformation.

## Methods

In this cross-sectional study, we used data from NewsGuard,^5^ a company that assesses the credibility and transparency of news websites using journalistic criteria, to identify news websites that repeatedly publish false or egregiously misleading content and were identified for publishing health misinformation as of August 2025 and topics on which those websites focus. We used data from MediaRadar, a company that produces commercial advertising intelligence, to estimate 2021 to 2024 advertising expenditures associated with each website identified by NewsGuard.^6^ MediaRadar’s MediaRadar360 database provides advertising expenditures for specific digital media categories. This study followed the STROBE reporting guideline. The Yale University institutional review board determined that neither consent nor ethics review was required because the study does not involve human participants. Additional details are provided in the eMethods in Supplement 1.

We calculated total annual advertising expenditures for all websites across all digital media categories, overall and stratified by 8 government and health organization advertiser categories (eTable in Supplement 1), identifying each category’s top 5 advertisers. Data analysis was conducted using R Studio, version 4.4.2.

## Results

There were 1229 news websites identified by NewsGuard for publishing health misinformation, and advertising expenditures were available from MediaRadar360 for 11; advertising expenditure data for internet display and mobile web were available for all 11 news websites (100%), online video for 2 (18%), mobile app for 1 (9%), and mobile web video for none. Content areas of significant focus included political news and commentary (n = 8), conspiracy theories or hoaxes (n = 5), health or medical information (n = 3), and general news (n = 2).

Overall, advertising expenditures from 2021 to 2024 for these 11 websites totaled $336 428 230, with $35 749 961 (10.6%) from government and health organizations. The median (IQR) advertising expenditure total per website among all advertisers and among government and health organizations was $13 448 278 ($10 656 148-$23 338 017) and $1 389 294 ($1 107 465-$2 162 362), respectively. Two websites, NewsMax and ZeroHedge, received 65.2% of all expenditures and 67.3% of expenditures by government and health-related organizations. The median (IQR) percentage of total advertising expenditure from government and health organizations per website was 9.7% (8.8%-11.2%), with 1 outlier (Healthy and Natural World) at 25.7%. Among government and health organization advertiser categories of interest, expenditures ranged from $571 843 on behalf of medical and health insurance companies to $19 242 621 on behalf of nonprescription remedy and wellness product advertisers (Table) and decreased from $16 688 593 in 2021 to $6 784 340 in 2024 (Figure).

**Table.** Estimated Advertising Expenditures From Government and Health Organizations to 11 News Websites Identified for Publishing Health Misinformation and the Top 5 Advertising Entities in Each Category, 2021-2024

Advertiser category	Expenditures (total $336 428 230)		Top 5 advertisers
	Advertising, $	% Among all advertisers	
Government and health organizations overall	35 749 961	10.6	City Beauty ($1 815 268) Gundry MD ($1 723 909) Boston Brain Science ($1 170 879) Walgreens ($1 168 387) Arthrozene ($1 082 354)
Nonprescription remedies and wellness products	19 242 621	5.7	City Beauty ($1 815 268) Gundry MD ($1 723 909) Boston Brain Science ($1 170 879) Arthrozene ($1 082 354) Swissklip ($1 002 585)
Medical service providers	6 933 023	2.1	Walgreens ($1 168 387) Seize the Awkward ($903 189) RexMD Drug Store ($883 941) Dentrix Enterprise ($255 690) Nuvia Dental Implant Center ($177 957)
Medical appliances, equipment, and devices	4 220 488	1.3	Aidion ($678 171) Thermo Fisher Scientific ($331 503) Smith & Wesson ($316 779) Scican STATIM ($223 155) Navage ($172 952)
Nonprofit medical and health organizations	1 669 064	0.5	Alzheimer’s Association ($89 395) American Heart Association ($66 794) College of American Pathologists ($30 490) Children’s Cancer Research Fund ($21 883) National Institute for the Clinical Application of Behavioral Medicine ($21 015)
Pharmaceuticals	1 352 710	0.4	Pfizer ($366 079) Eli Lilly ($257 676) Sanofi ($235 890) Avanir Pharmaceuticals ($110 428) Allergan ($74 980)
US, state, and local government (non-HHS)	1 137 201	0.3	Peace Corps ($598 416) Navy Exchange Store ($161 212) National Highway Traffic Safety Administration ($89 087) US Air Force ($78 025) Social Security Administration ($35 490)
HHS and subsidiaries	623 013	0.2	Centers for Medicare & Medicaid Services ($319 743)^a^ HHS ($171 592) Centers for Disease Control and Prevention ($114 446) Vaccines.gov ($9696) US Food and Drug Administration ($7535)
Medical and health insurance	571 843	0.2	Mutual of Omaha ($617 652) Blue Cross Blue Shield of North Carolina ($258 339) Blue Cross Blue Shield ($210 023) Humana ($131 035) Kaiser Permanente ($47 834)

Abbreviation: HHS, US Department of Health and Human Services.

^a^ Expenditure totals for Centers for Medicare & Medicaid Services, Healthcare.gov, and Medicare were combined.

**Figure.**
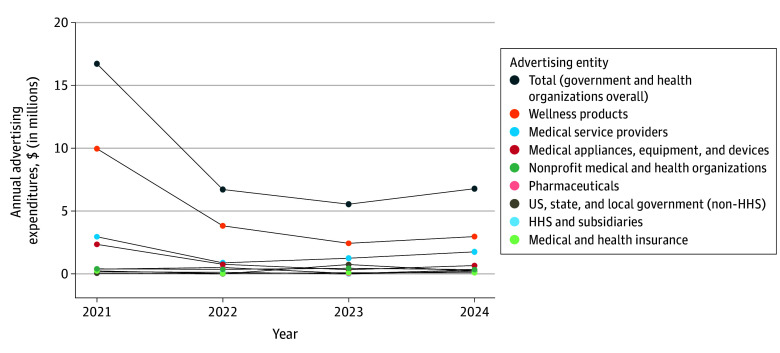
Estimated Advertising Expenditures From Government and Health Organizations to 11 News Websites Identified for Publishing Health Misinformation, 2021-2024 Each dot represents the estimated annual total advertising expenditure from advertisers in the associated advertising category to place digital advertisements on 11 news websites that were identified by NewsGuard to have published health misinformation. Advertising expenditure estimates are from MediaRadar. HHS indicates US Department of Health and Human Services.

## Discussion

From 2021 to 2024, government and health organizations accounted for about one-tenth of the $336 million in estimated advertising payments made to 11 news websites identified for publishing health misinformation. Noteworthy advertisers included federal health agencies, such as the Centers for Disease Control and Prevention, and pharmaceutical companies, such as Pfizer, although their individual contributions represented a small share of all payments.

Although our study was limited to only 11 news websites identified for publishing health misinformation with available advertising expenditure data, and the proportion of website material made up of misinformation is not known, our findings illustrate the possibly inadvertent financial support many government and health organizations are providing by making advertising payments to these news websites. Advertisements from these organizations may enhance trust in misinformation or diminish trust in the government or health organization. Stronger restrictions may be needed to avoid placing advertisements on news websites that spread health misinformation.

## Notes

Supplement 1.
eMethods.

eReferences.
**eTable.** Government and Health Organization Advertiser Categories and Descriptions

Supplement 2.
Data Sharing Statement
