# Supplementary material for: Advertising Payments to News Websites That Publish Health Misinformation
Source: JAMA Netw Open. 2026 Apr 1;9(4):e265068. doi: 10.1001/jamanetworkopen.2026.5068 (PMC13044671; doi:10.1001/jamanetworkopen.2026.5068)
Supplement: Supplement 1. — eMethods. eReferences. eTable. Government and Health Organization Advertiser Categories and Descriptions [file jamanetwopen-e265068-s001.pdf]

# Supplemental Online Content

Patel NG, Ramachandran R, Ross JS. Advertising payments to news websites that publish health misinformation. *JAMA Netw Open*. 2026;9(4):e265068. doi:10.1001/jamanetworkopen.2026.5068

**eMethods.**

**eReferences.**

**eTable.** Government and Health Organization Advertiser Categories and Descriptions

This supplemental material has been provided by the authors to give readers additional information about their work.

**eMethods.**

## Generating sample of websites

To create our sample of websites identified for publishing health misinformation, we used the Media Intelligence Dashboard from NewsGuard, a company that produces data, analysis, and journalism reports related to the reliability of online information. The Media Intelligence Dashboard includes quality ratings of news websites based on assessments from a team of expert journalists that review each website. The database was chosen because its ratings are periodically updated, use transparent and apolitical criteria,[1] and have been used in previous research to identify websites that produce misinformation.[2] We identified news websites that, as of August 7, 2025 had failed NewsGuard's criterion "does not repeatedly publish false or egregiously misleading content" and were flagged by NewsGuard's team for recently publishing health misinformation, defined as a provably false or egregiously misleading claim that is significantly related to a health topic, i.e. a cure or treatment for a disease, information related to a disease outbreak, vaccine, or medical product, or health-related legislation or policy.

We used the advertising data platform MediaRadar360 to identify website "properties" (i.e. websites that have been tracked for advertising data) with available digital advertising data. MediaRadar360 is a service of MediaRadar, a company that provides commercial advertising intelligence to advertisers. The MediaRadar360 database includes estimates of digital advertising expenditures made to properties based on estimates of price metrics and ad impressions.[3] MediaRadar360 includes all website domains for which there was any available advertising expenditure data, not limited to news websites. MediaRadar360 assigns digital advertising expenditures to several categories of which we included the following: internet display, online video, mobile app, mobile web, and mobile web video. We included in our final sample any website identified to publish health misinformation that also had associated digital advertising expenditure data available in MediaRadar360 for our study period.

## Generating advertising expenditure dataset

We used the MediaRadar360 platform to generate a report of annual digital advertising expenditure data from 2021-2024 by website property, that was further delineated by advertiser. The report also included information about each advertiser's industry category and subcategory.

We removed all entries for which the advertiser was listed as unknown. When data for a digital media category was available for a website, it was available for all four years of the study period.

We used MediaRadar360's advertiser category classifications to identify advertising payments that were from eight government and health organization categories of interest: Nonprescription Remedies and Wellness Products; Medical Service Providers; Medical Appliances, Equipment, and Devices; Nonprofit Medical and Health Organizations; the U.S. Department of Health and Human Services and its subsidiaries; Federal, State, and Local Government (Non-HHS); Pharmaceuticals; and Medical & Health Insurance. Further details are included in eTable.

**Data analysis**

We calculated total advertising expenditure from all known advertisers, as well as from advertisers categorized as government or health organizations, overall and for each year of our study. We also calculated the percentage of total advertising expenditures that came from each government and health organization category.

At the website level, we calculated the median and IQR of total advertising expenditures and advertising expenditures from government and health advertisers, as well as the percentage of each website's total advertising expenditures that came from government and health organizations.

**eReferences.**

1. NewsGuard. Website Rating Process and Criteria. Accessed September 17, 2025. https://www.newsguardtech.com/ratings/rating-process-criteria/

2. Ahmad, W., Sen, A., Eesley, C., & Brynjolfsson, E. Companies inadvertently fund online misinformation despite consumer backlash. *Nature*, 2024;630(8015), 123-131. https://doi.org/10.1038/s41586-024-07404-1

3. MediaRadar360 Support. MediaRadar360 Methodology. Accessed September 17, 2025. https://intercom.help/vivvix-360/en/articles/8562801-mediaradar-360-methodology-summary

© 2026 Patel NG et al. *JAMA Network Open.* 

**eTable.** Government and Health Organization Advertiser Categories and Descriptions

| **Parent Category** | **Description** |
|---|---|
| Nonprescription Remedies and Wellness Products | Non-prescription Remedies; Personal Care products; Fitness & diet programs; Spas |
| Medical Service Providers | Hospitals, Clinics, Medical Centers; Retirement and Nursing Homes, Doctors and Dental Offices, Drugstores and Pharmacies |
| Med Appliances, Equipment, & Devices | Medical Appliances, Equipment, and Devices |
| Nonprofit Medical & Health Organizations | Nonprofit Medical and Health Organizations |
| Pharmaceuticals | Pharmaceuticals |
| US, State, & Local Government (Non-HHS) | US Government (excluding HHS and its subsidiaries), Local and State Government |
| HHS and subsidiaries | Department of Health and Human Services and its subsidiaries |
| Medical & Health Insurance | Medical and Health Insurances companies; Healthcare Maintenance Organizations |
