# Supplementary material for: Advertising Payments to News Websites That Publish Health Misinformation
Source: JAMA Netw Open. 2026 Apr 1;9(4):e265068. doi: 10.1001/jamanetworkopen.2026.5068 (PMC13044671; doi:10.1001/jamanetworkopen.2026.5068)
Supplement: Supplement 2. — Data Sharing Statement [file jamanetwopen-e265068-s002.pdf]

Data Sharing Statement

Patel. Advertising Payments to News Websites That Publish Health Misinformation. *JAMA Netw Open*. Published April 01, 2026. doi:10.1001/jamanetworkopen.2026.5068

Data
Data available: No

Additional Information
Explanation for why data not available: The data used for this study are owned by the organization NewsGuard and can only be obtained via license.
